# Lessons from Real Life Experience: Importance of In-House Sequencing and Smart Ratio-Based Real-Time PCR Outperform Multiplex Ligation-Dependent Probe Amplification in Prenatal Diagnosis for Spinal Muscular Atrophy: Bench to Bedside Diagnosis

**DOI:** 10.1055/s-0043-1774307

**Published:** 2023-08-31

**Authors:** Gulten Tuncel, Burcin Sanlıdag, Eray Dirik, Tugba Baris, Mahmut Cerkez Ergoren, Sehime Gulsun Temel

**Affiliations:** 1 Department of Medical Genetics, Faculty of Medicine, Near East University, Nicosia, Cyprus; 2 DESAM Research Institute, Near East University, Nicosia, Cyprus; 3 Department of Paediatrics, Faculty of Medicine, Near East University, Nicosia, Cyprus; 4 Gelişim Tıp Laboratuvarları, Istanbul, Turkey; 5 Department of Medical Genetics, Faculty of Medicine, Bursa Uludag University, Bursa, Turkey; 6 Department of Translational Medicine, Institute of Health Science, Bursa Uludag University, Bursa, Turkey; 7 Department of Histology and Embryology, Faculty of Medicine, Bursa Uludag University, Bursa, Turkey

**Keywords:** SMA, *SMN1*, MLPA, sequencing, in-house testing

## Abstract

Spinal muscular atrophy (SMA) is a rare, recessively inherited neurodegenerative disorder caused by the presence of pathogenic variants in the *SMN* gene. As it is the leading inherited cause of infant mortality, identification of *SMN* gene pathogenic variant carriers is important for diagnostic purposes with effective genetic counseling. Multiple ligation probe analysis (MLPA), a probe-based method, is considered as the gold standard for SMA carrier analysis. However, MLPA might give false-negative results in cases with variations in the probe-binding regions. Here, we present a case born to consanguineous SMA carrier parents. Prenatal diagnosis with MLPA failed to detect the compound heterozygous mutant state of the proband and she was born unfortunately with SMA phenotype. Further analysis with a real-time polymerase chain reaction kit was able to detect the compound heterozygous state of the patient and was confirmed with targeted next-generation sequencing technology.

## Introduction

Spinal muscular atrophy (SMA) is a rare, recessively inherited neurodegenerative disorder arising from deterioration of motor neurons in the anterior horn of the spinal cord that in turn affect voluntary muscle movements resulting in muscle weakness and atrophy. 1 The prevalence of SMA is estimated to be 1 in 10,000 live births worldwide. SMA has very diverse clinical phenotypes and is classified on the basis of age of onset and clinical symptom severity. Congenital SMA is usually represented with neonatal hypotonia, respiratory failure, and immobility, which results in infant morbidity within the first month postmortem. 2 SMA type I (Online Mendelian Inheritance in Man [OMIM] 253300) is the most severe form with very early-onset symptoms starting in the first 6 months of life. About 45% of the cases are represented with hypotonia, aflexia, and weakened diaphragmatic function leading to bell-shaped chest. Without proper ventilation aid most of the carriers of this type could die within two. 3 4 Type II (OMIM 253550) is the intermediate form presented within patients 6 to 18 months old and the symptoms are similar to type I with the addition of joint contracture and proximal weakness occurring in the legs. Majority of patients can survive till the third decade. Type III (OMIM 253400) patients have the least severe symptoms that develop after 18 months of age, whereas type IV SMA (OMIM 271150) or what is known as adult SMA is known to affect the adults and only causes mild symptoms. 5 6

SMA is caused by pathogenic variations in the telomeric copy of the *SMN* gene, referred as *SMN1* ( *SMN1* ; OMIM 600354), located on chromosome 5q13. Encoded survival motor neuron (SMN) protein is a part of a multiprotein complex found in the cytoplasm and nucleus of various cell types including the motor neurons with significant roles in ribonuclease protein biogenesis and pre-ribonucleic acid splicing. 7 8 The most frequent pathogenic variations in the *SMN1* gene are the deletion in exon 7 or deletion in both exons 7 and 8, which generally results in the synthesis of a nonfunctional or a truncated protein with insufficient function. Reduced amounts of functional SMN protein leads to the deterioration of motor neurons causing muscle weakness and atrophy. 9 Other least frequent variations in the *SMN1* gene are point mutations. Absence of *SMN1* can partially be compensated by *SMN2* ( *SMN2* ; OMIM 600354), which is the centromeric copy of *SMN1* , with 99% nucleotide identity created by an inverted duplication on the chromosome. However, as a large majority of *SMN2* transcripts lack exon 7 due to the c.840C > T substitution, only a small amount of normal, functional SMN proteins can be produced. 1 A study by Mailman et al revealed that the majority of patients identified with SMA had insufficient *SMN1* gene production, thereby reliant on their *SMN2* gene production. They also clarified that higher copy numbers in *SMN2* gene is directly associated with mild symptoms of the disease. As without them loss of function in *SMN1* gene is extremely fatal. 10 Initially, diagnosis was only performed by clinical tests like creatine kinase, nerve conduction study, muscle biopsy, and magnetic resonance imaging. 11 Later, molecular testing technologies have become the standard methods for the diagnosis of SMA due to their high efficiency and specificity.

Previous investigation on the treatment of SMA was mainly focused on treating the symptoms and reused pharmaceutical compounds with positive outcomes on other illnesses to address primary and secondary symptoms of SMA. 12 Nowadays, therapeutic approaches and trials are focusing on to upregulate muscle growth, modify the splicing of *SMN2* , or replace the *SMN1* gene. The first successful genetic therapy was conformed in 2010 to 2011 through the replacement of *SMN1* gene in murine mouse models and the use of AVXS-101 (onasemnogene abeparvovec, Zolgensma) that was approved by the Food and Drug Administration for SMA patients under the age of 2 in May 2019. 13 14 15 However, as the treatment is still very expensive, the drug is not obtainable for most patients. Therefore, SMA diagnostic tests like prenatal chorionic villus sampling and amniocentesis as well as preimplantation embryogenic testing during assisted reproductive technology-in vitro fertilization are of high importance to reduce the burden of having an affected individual for families.

Currently, SMA screening tests are done with multiple ligation probe analysis (MLPA) and are widely considered as the gold standard. However, in some cases MLPA analysis can be inconclusive and/or insufficient for diagnosis and other techniques are required for confirmation. Among these, targeted gene sequencing has been a powerful alternative. In recent years, on the other hand, faster and cheaper alternatives such as multiplex real-time polymerase chain reaction (PCR) and digital droplet PCR (ddPCR) kits were developed and are in use for SMA screening. 16

Here, we present a case where the compound heterozygote state of variations could not be detected with prenatal MLPA screening which was purchased from another laboratory by the gynecologist. Unfortunately, diagnosis was possible after the birth with targeted gene sequencing and real-time PCR (SNP Biotechnology) which has been done from bench to bedside diagnosis in our laboratory.

## Case Presentation and Study Design

A 3-month-old female patient was admitted to the Near East University Department of Child Neurology Outpatient Clinic with complaints of muscle weakness that was recognized from birth, difficulty in moving her foot, inability to hold her head up, and accompanying respiratory problems. The diminished movements were mostly recognized on her hands and arms. She was born term,3,600 g of weight, and she had a newborn intensive care admission of 1-week period because of respiratory distress. The parents also dedicated a progressively increasing difficulty in feeding from birth.

**Fig. 1:**
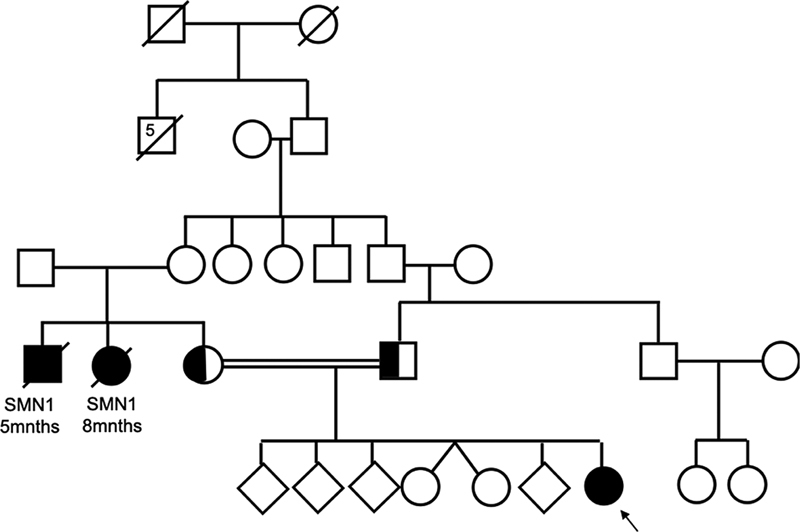
Family pedigree of the proband, presented with an arrow, is shown in this figure. Parents were second-degree relatives with a familial history of two exitus with the diagnosis of spinal muscular atrophy (SMA) (mother's siblings). She was the third live birth of seventh pregnancy of the mother with five abortus and twin healthy girl siblings.

She was the third live birth of seventh pregnancy of the mother with five abortus and twin healthy girl siblings. Parents were second-degree relatives with a familial history of two exitus with the diagnosis of SMA (mothers' siblings). Family pedigree is shown in Fig. 1 . Previous analyses have shown that both parents were heterozygous carriers for pathogenic *SMN1* gene variations. The mother has a known heterozygote deletion in *SMN1* exon 7 and the father has a heterozygote deletion in *SMN1* exons 7 and 8. Prenatal genetic analysis with MLPA to detect small *SMN1* and *SMN2* exon insertion-deletion variations reported heterozygous *SMN1* exon 7 and 8 deletion in the proband. No deletion or duplication was reported in the *SMN2* gene.

In her physical examination vital signs were within normal limits. Her weight, height, and head circumference were 4.920 g (10–25 percentile), 58 cm (75–90 percentile), and 38.8 cm (50 percentile), respectively. She had eye contact and social smiling. The head control was poor, and she had fasciculation on her tongue. Deep tendon reflexes were absent. The rest of the examination was unremarkable. The electromyelography revealed a subacute/chronic cervical (C5-6, T1) and lumbosacral (L3-S1) involvement of spinal anterior horn cells.

**Fig. 2:**
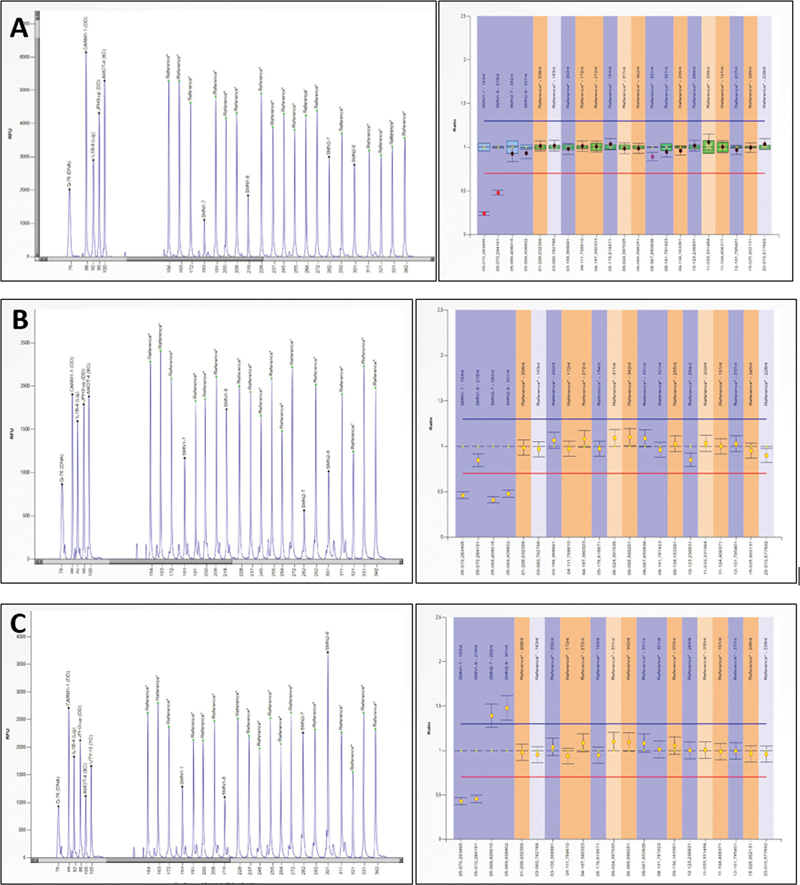
Multiple ligation probe analysis (MLPA) results of the proband ( **A** ), mother ( **B** ), and the father ( **C** ) are represented in this figure. Heterozygous deletion in *SMN1* exon 7 and 8 is detected in the proband, likely inherited from her father. The probe amplification pattern of the proband is in concordance with her father's.

When the patient was admitted to our hospital with these symptoms associated with SMA type I, MLPA analysis was repeated with SALSA MLPA Probemix P060 SMA Carrier assay (MRC-Holland), using deoxyribonucleic acid (DNA) isolated from peripheric blood samples of the proband and the parents. MLPA results showed intact exon 8 and heterozygote deletion of exon 7 in the mother, and heterozygote exon 7 and 8 deletion in the father. However, the results were inconclusive for the proband ( Fig. 2 , MLPA after birth).

**Fig. 3:**
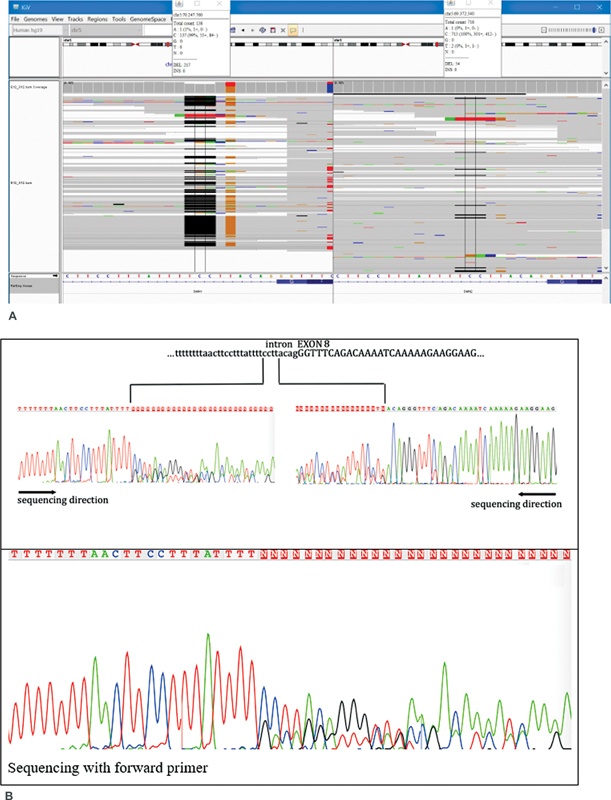
( **A** ) Targeted *SMN1* (left panel) and *SMN2* (right panel) gene sequencing results of the proband is represented in this figure. ( **B** ) Confirmation of the sequencing results of the variation detected on exon 8 with Sanger sequencing is given in this figure.

As MLPA results were inconclusive and not supporting the patient's phenotype and clinic, further analysis with targeted *SMN1* gene sequencing was performed in Illumina MiSeq platform. Sequencing results indicated a heterozygous c.835-5_835-9delTCCTTinsTG (IVS7-5_IVS7-9delTCCTTinsTG) variation in the mother, which was inherited to the proband ( Fig. 3A , next-generation sequencing [NGS]). Proband was found to be compound heterozygote in exon 7 and heterozygote for exon 8 deletion. NGS results were further confirmed with Sanger sequencing ( Fig. 3B , Sanger).

**Fig. 4:**
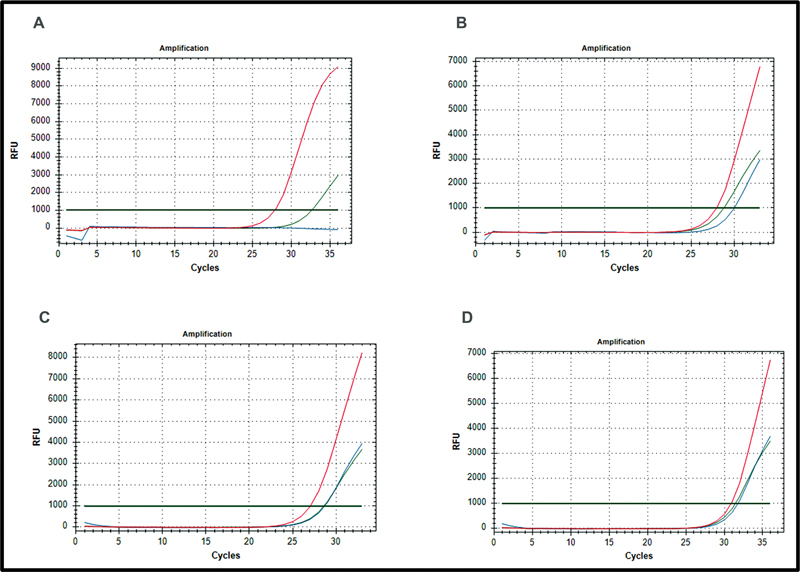
Analysis results of the proband ( **A** ), mother ( **B** ), father ( **C** ), and a wild-type sample ( **D** ) by spinal muscular atrophy (SMA) qPCR Detection Kit by SNP Biotechnology (Cat 200R-10-01) is represented in this figure. Homozygous mutant state of the proband is detected by the kit as according to the manufacturer's instructions no signal in FAM and HEX channels indicates homozygous mutation carriers. Mother and father were evaluated as heterozygous carriers. Texas Red, internal control; FAM, *SMN1* ; HEX, *SMN2* .

In parallel to the targeted sequencing analysis, SMA qPCR Detection Kit by SNP Biotechnology (Cat 200R-10-01) was used to test the proband's sample. SNP SMA Screening Kit detects the exon 7 deletion with C/T substitution at nucleotide 840 and exon 8 deletion in the *SMN1* gene to diagnose the affected states by quantitative real-time PCR (qPCR) from blood. Compound heterozygote state of the patient, which was missed by MLPA analysis, could also successfully be detected by the qPCR ( Fig. 4 , real-time PCR).

## Discussion

SMA is an inherited neuromuscular disorder that is characterized by muscle weakness and atrophy due to progressive degeneration of the anterior horn cells of the spinal cord. History of motor difficulties, reduced deep tendon reflexes, and muscle weakness together with the presence of biallelic pathogenic variants in the *SMN1* gene in more than 90% of patients establish the SMA diagnosis. 3 Great majority of the SMA patients carry deletion mutations in their *SMN1* exon 7 leading to the production of a nonfunctional protein product and changes in the centromeric *SMN2* gene copy number is known to modify the phenotype of patients as a small amount of functional SMN protein is produced by the *SMN2* to compensate the pathogenic loss of the protein by *SMN1* deletion mutations. 17 The high sequence similarity between *SMN1* and *SMN2* genes can be problematic in disease and carrier screening tests.

As one of the leading inherited causes of infant mortality, identification of carriers for the *SMN* gene mutations is important for diagnostic purposes and for effective genetic counseling. Molecular diagnostic methods that are used for SMA patient and carrier testing include restriction fragment length polymorphism (RFLP) test, MLPA, qPCR, and DNA sequencing. 18 RFLP test has already been replaced by advanced technologies including MLPA, targeted sequencing, and multiplex qPCR, and is not in use in routine laboratories. Moreover, currently ddPCR is being employed in SMA analysis, which will further reduce cost, time, and labor.

In the current case, prenatal and postnatal tests were performed using the MLPA technique, which is often considered as the gold standard for SMA diagnosis, providing an easy, fast, and high-throughput system for analyzing the SMA critical region both in affected patients and in healthy carriers. 19 20 The technique depends on the ligation of mutation-specific probes to the *SMN* genes to create a reading in presence of a mutation. However, point mutations or less frequent mutations in the probe-hybridization loci such as the deletion/insertion mutation detected in our patient (c.835-5_835-9delTCCTTinsTG (IVS7-5_IVS7-9delTCCTTinsTG)) can cause inconclusive results as seen in our MLPA analysis. In a study by Sharifi et al, where 150 unrelated families with at least one suspected SMA patient in their family were surveyed for *SMN* gene, they showed false-negative carrier results where individuals had point mutations in the gene and also that heterozygous healthy carriers with two copies of *SMN1* in one chromosome may not be identified by the MLPA analysis. 21 Therefore, for prenatal diagnostic, newborn and carrier screening just with this method in such families is risky, at least screening should be done with an additional method, especially for prenatal testing.

In this context, it is important to consider additional and alternative testing methods that can also reduce cost and have increased availability in the laboratory, including in-house designed real-time PCR technology. *SMN1* targeted sequencing results of our proband's sample clearly revealed the mutation present in a heterozygous state with exon 7 and 8 deletion inherited from the father. In a previous study, a SMA patient with c.835-5T > G variation was reported, which cause the exclusion of exon 7. 22 A 7-base pair deletion in the same gene region was also reported by Melki et al. 23 c.835-5_835-9delTCCTTinsTG (IVS7-5_IVS7-9delTCCTTinsTG) was considered pathogenic with a high probability according to the American College of Medical Genetics and Genomics criteria. 24 Considering the patient's MLPA report, compound heterozygosity is the likely explanation of the patient's SMA phenotype. Compound heterozygote mutant state of our patient could also be detected by SMA qPCR Detection Kit by SNP Biotechnology (Cat 200R-10-01). Although it is not possible to detect the precise change in the DNA sequence, being cost effective, less labor-intensive, and as a widely available technology in the laboratories, use of real-time PCR kits is a valuable alternative to targeted sequencing.

Overall, it is clear that SMA carrier screening tests are of great importance to reduce the burden of having an affected child for families. Even though MLPA is the most frequently used technique for testing and is considered as the gold standard, it can be insufficient in cases where the individual carries nonfrequent or point mutations in probe-binding regions. In this context, we argue that application of prenatal testing with two methods especially in cases with a family history and also in data with inconclusive results is beyond critical. We also would like to point out the importance of in-house genetic testing from bench to bedside diagnosis instead of purchasing genetic tests from external genetic test providers without seeing the results and analysis.
